# Defensive functions and potential ecological conflicts of floral stickiness

**DOI:** 10.1038/s41598-022-23261-2

**Published:** 2022-11-18

**Authors:** Alexander Chautá, Arvind Kumar, Jesica Mejia, Elena E. Stashenko, André Kessler

**Affiliations:** 1 Department of Ecology and Evolutionary Biology, Cornell University, Ithaca, NY USA; 2 Center for Chromatography and Mass Spectrometry, CROM-MASS, CIBIMOL-CENIVAM, Industrial University of Santander, Carrera 27, Calle 9, Edificio 45, 680002 Bucaramanga, Colombia; 3 Centre for Aromatic Plants (CAP), Industrial Estate, Selaqui, Dehradun, Uttarakhand 248 011 India

**Keywords:** Ecology, Community ecology, Tropical ecology

## Abstract

Stickiness of vegetative tissues has evolved multiple times in different plant families but is rare and understudied in flowers. While stickiness in general is thought to function primarily as a defense against herbivores, it may compromise mutualistic interactions (such as those with pollinators) in reproductive tissues. Here, we test the hypothesis that stickiness on flower petals of the High-Andean plant, *Bejaria resinosa* (Ericaceae), functions as a defense against florivores. We address ecological consequences and discuss potential trade-offs associated with a repellant trait expressed in flowers that mediate mutualistic interactions. In surveys and manipulative experiments, we assess florivory and resulting fitness effects on plants with sticky and non-sticky flowers in different native populations of *B. resinosa* in Colombia*.* In addition, we analyze the volatile and non-volatile components in sticky and non-sticky flower morphs to understand the chemical information context within which stickiness is expressed. We demonstrate that fruit set is strongly affected by floral stickiness but also varies with population. While identifying floral stickiness as a major defensive function, our data also suggest that the context-dependency of chemical defense functionality likely arises from differential availability of primary pollinators and potential trade-offs between chemical defense with different modes of action.

## Introduction

**Figure 1:**
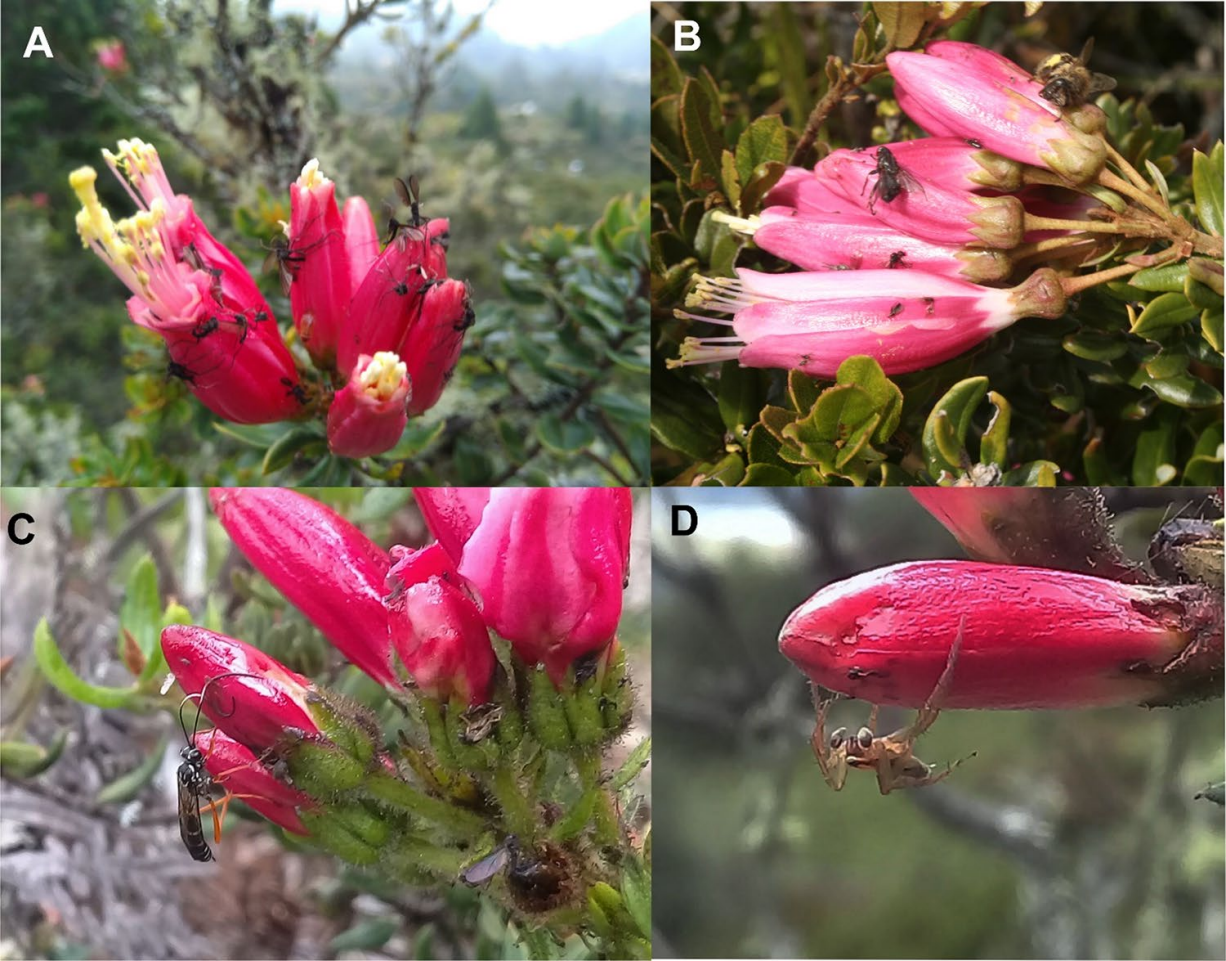
Insects caught on sticky flowers of *Bejaria resinosa.* (**A**) Multiple flies, (**B**) Bee, (**C**) Wasp, (**D**) Spider.

Plants have evolved multiple strategies to cope with herbivores. These strategies range from morphological defenses (e.g. thorns and trichomes) over tolerance to a complex set of secondary metabolites that can mediate direct and indirect resistance and repellency. Direct defensive secondary metabolites function through different mechanisms and include antinutritive, antidigestive, repellent, or physical–chemical hybrid traits. Physical–chemical hybrid defenses include surface waxes^1^, latex^2^, resin^3^, and sticky compounds exuded from trichomes^4^ or epidermis cells that prevent insects from accessing the plant tissues surface or compromise mobility (Fig. 1). The latter, sticky compounds, are particularly interesting for their interesting ecological and evolutionary implications, which are poorly studied. The stickiness of plant vegetative surfaces is not rare and can be found in a diversity of species across different plant families^5^. Sticky compounds are most commonly exuded by glandular trichomes, but also by specialized glands that produce mucilage and compounds like acyl sugars, terpenoids, phenolics, and ketones^6,7^. Several functional hypotheses on the production of leaf surface adhesive compounds have been proposed.

First, sticky compounds on leaf surfaces can function as a direct defense and repel or kill antagonists, such as herbivores. Sticky plants can trap herbivores and kill them by starvation^8^ or the adhesive can include toxic substances and so affect antagonists that get stuck on or ingest those compounds. For example, sesquiterpenes found in glandular trichomes of wild tomato species have been found to increase the mortality of Colorado potato beetle^9^. Also, the presence of glandular trichomes on *Datura wrightii* was found to reduce the performance of *Manduca sexta* by increasing the time to pupation and reducing consumption rates^10^.

Second, arthropods trapped on sticky plant surfaces may attract predators that then further reduce herbivore pressure on the plant^5^. In such cases, compounds mediating the adherence of prey organisms and so facilitating the residence of predators can be viewed as indirect defense traits^11^. In some cases, the number of predators on sticky plants was found to proportionally increase with the number of insects stuck to the plant^12^. The attraction of predators can reduce herbivore abundances and thus herbivory beyond the direct defensive effects of sticky compounds with potential additive or synergistic effects on plant fitness^5,13^. A similar indirect defensive function of sticky compounds can be assigned in cases where volatile breakdown products of sticky compounds ingested by herbivores can function as prey-finding cues. For example, *O*-acyl sugars produced by trichomes of the wild tobacco *Nicotiana attenuata* are converted into a volatile branched-chain aliphatic acid in the guts of *Manduca sexta* caterpillars*.* An omnivorous ant, *Pogonomyrmex rugosus,* has been found to use this volatile cue to locate its caterpillar prey^6^. In addition, the presence of insects glued to the petals could attract bird pollinators that opportunistically forage for arthropods to fulfill their protein requirements.

Third, stickiness can help plants with acquiring additional nutrients^14^. Soil nitrogen availability is limited in many natural soil types. However, in particularly limited habitats, plants have evolved ways of acquiring alternative resources of nitrogen, such as from captured animals. This strategy is notorious for carnivorous plants, but it is also found in a few sticky plants without specified prey-capture organs. For example, *Geranium viscosissimum*, *Potentilla arguta,* and *Stylidium* sp. can partially digest the proteins of insects trapped on their glandular trichomes^15,16^. While these plants are capturing and digesting insects, they are not considered carnivorous but rather protocarnivorous, because the primary role of stickiness is assumed not to be capturing prey for supplemental nutrition^17^. To our knowledge, a thorough cost–benefit assessment of the different ecological functions of stickiness in plants has not yet been conducted.

Despite the seemingly obvious benefits of sticky compounds on leaf surfaces^18^, there are also some associated ecological costs^18^. For example, sticky surfaces can trap dust that could potentially affect light availability for photosynthesis^19^. In addition, mutualists could be affected by stickiness with potential direct negative effects to plant fitness^19^. Such ecological costs are probably the reason for the fact that while stickiness is a common trait of plant vegetative tissues, it is much less common in flowers, specifically petals. Ultimately, flowers are tissues that have evolved to facilitate interactions with mutualists. On the other hand, out of all the plant tissues, the herbivore damage to the flowers can be the most deleterious. Herbivores can reduce plant fitness significantly by imposing even minute amounts of damage to reproductive parts (e.g. anthers, style, ovaries) or, when damaging corolla or colored bracts by changing the information transfer between plants and pollinators^20,21^. This is hypothesized as a reason why floral tissues are usually found to be more strongly constitutively defended with secondary metabolites (e.g. phenolic compounds, glycosides, soluble myrosinases)^22–24^ and deterrent volatiles^25,26^ than leaf tissues.

However, the rarity of stickiness in petals is likely due to the more direct negative effects on potential pollinators. Stickiness can trap insect pollinators reducing plant pollination and fitness. More importantly to this study, the stickiness can also affect interactions of the plant with other mutualists, by, for example, entrapping small parasitoids^27^ or reducing predator mobility^27^, thus compromising indirect defenses. Because of this more directly played out conflict between repelling antagonists and attracting mutualists, interactions of plants that feature sticky floral parts with their interacting community provide ideal models to understand the ecological dynamics of conflicting interactions mediated by plant chemistry. For example, the existence of such an ecological conflict mediated by sticky chemistry has recently been suggested by a study of *Erica* spp. in South Africa. Across the *Erica* species, floral stickiness excluded most insect herbivores and was associated with long-proboscid fly and bird pollination while it mediated significant protection against nectar robbers^28^. Nevertheless, floral stickiness remains poorly studied both functionally (including basic anti-florivore defense function) and mechanistically^28^.

*Bejaria resinosa* Mutis ex L. (Ericaceae) is an up to 3 m tall shrub with red–purple flowers native to the poorly studied High-Andean Paramo and sub Paramo ecosystems of Venezuela, Ecuador, Perú, and Colombia between 1750 and 3700 m.a.s.l.^29^. As many other species in the genus *Bejaria*, the flowers of this species are sticky. However, the most distinctive trait of *B. resinosa* is that flowers vary in stickiness between individuals^30,31^. Moreover, populations vary in the number of individuals with sticky versus non-sticky flowers, making it also a great model for understanding the rarely tested ecological functions of stickiness in plants. Remarkably, stickiness in this species is present exclusively in petals and sepals and not in other tissues of the plant. The main pollinators of *B. resinosa* are bumble bees and hummingbirds^30^. Petals of sticky flowers are usually loaded with a diversity of trapped insects, which includes flies, bees, wasps, butterflies, and spiders, some of which could well function as pollinators^30^, which led previous researchers to speculate that stickiness can help these plants with acquiring additional nutrients^14^. However, the relatively large flowers are also attractive to herbivorous insects and different species of avian flower piercers of the genus *Diglossa* that are specialists in nectar-robbing. Here, we use a natural polymorphism in stickiness to address the hypothesis that floral stickiness in *B. resinosa* functions as a defense against florivores. We use field surveys and manipulative experiments to measure the effects of stickiness on herbivory, assess the effects on plant fitness, identify factors that influence the defense-functionality of stickiness (context-dependency) and begin to identify the chemical properties of sticky and non-sticky *B. resinosa* flowers.

## Results

### Floral stickiness correlates negatively with florivory and fruit set

**Figure 2:**
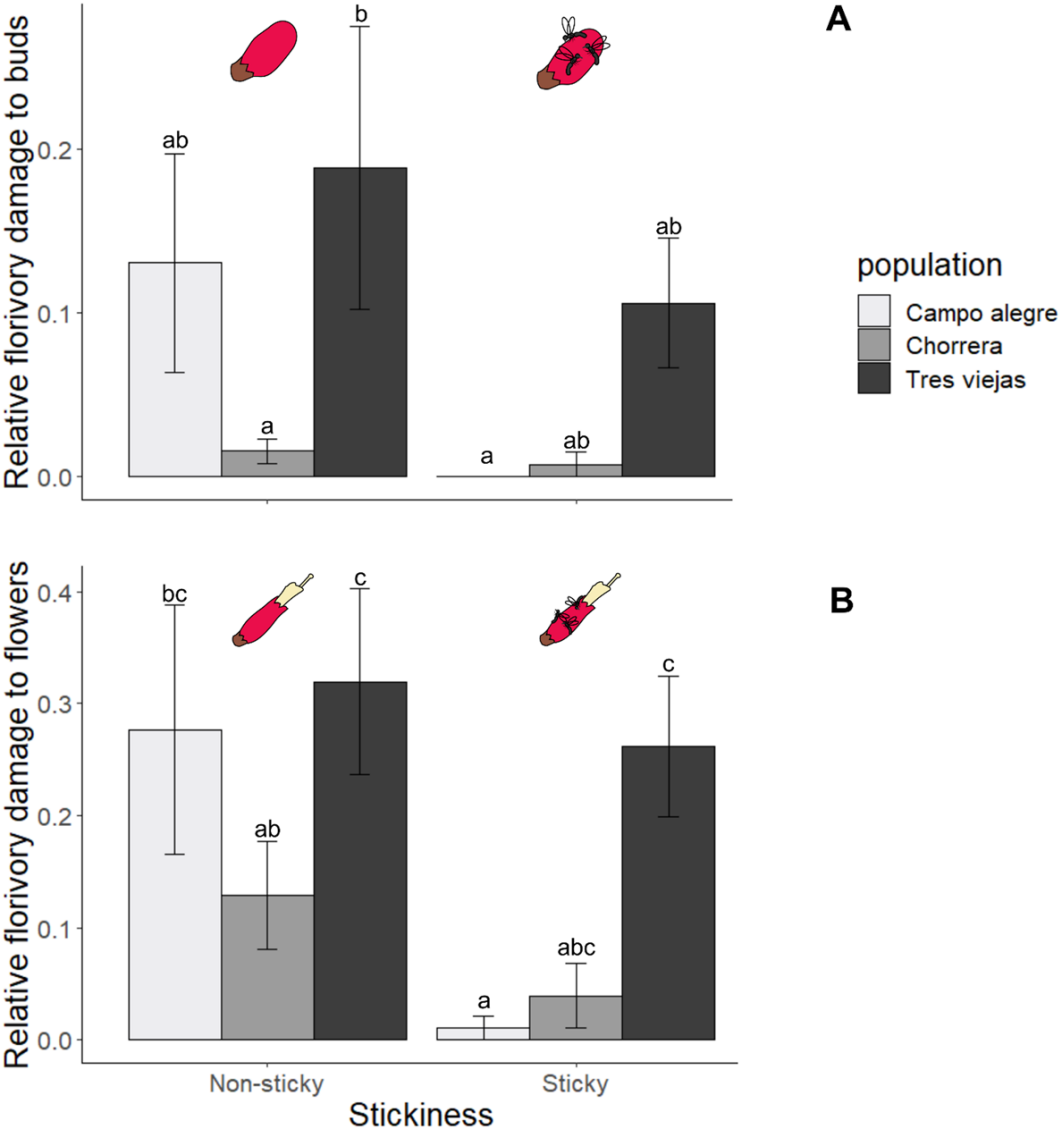
Mean relative proportion of buds (**A**) and flowers (**B**) (± SE) with damage by florivores on sticky and non-sticky plants of *Bejaria resinosa* in three different populations. Different letters indicate significant differences (Tukey test, significance *P* = 0.05).

The florivore damage to buds was higher on non-sticky flowers and varied with plant population (*Χ*^2^ = 12.007, *df* = 3, *P* = 0.007; *Χ*^2^ = 20.83, *df* = 4, *P* < 0.001, respectively). Because stickiness did not equally reduce herbivory in all three populations, stickiness and population interacted as factors (*Χ*^2^ = 6.1338, *df* = 2, *P* = 0.046, Fig. 2A).

Florivory had a prevalence of 14.09% across all populations. Stickiness and population both also affected the proportion of flowers damaged by florivores (*Χ*^2^ = 23.82, *df* = 3, *P* < 0.001; *Χ*^2^ = 46.895, *df* = 4, *P* < 0.001, respectively) with sticky flowers receiving less damage. Like the effects on buds, stickiness affected damage to open flowers differently in the different plant populations (*Χ*^2^ = 15.989, *df* = 2, *P* < 0.001, Fig. 2B).

**Figure 3:**
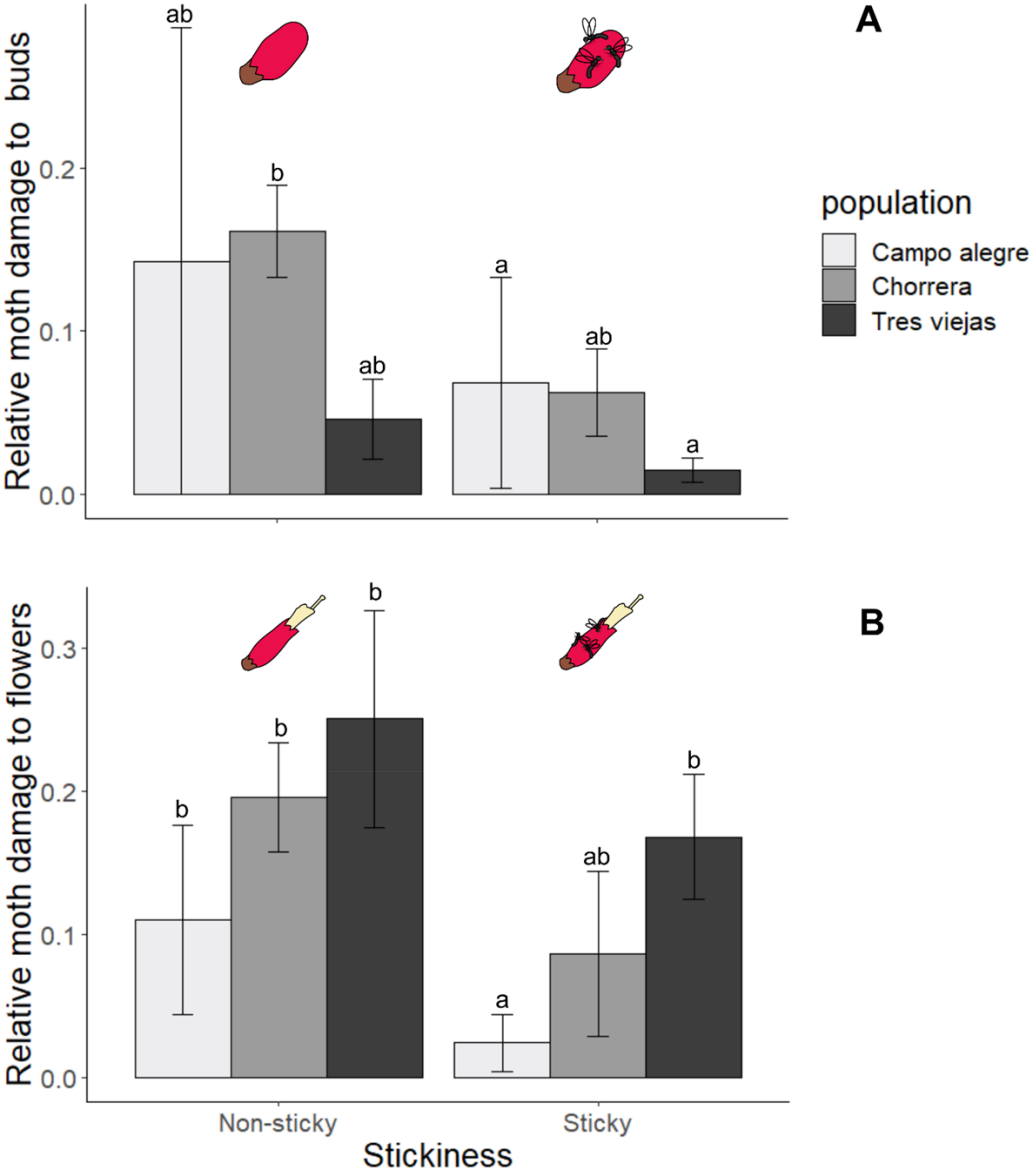
Relative amount of buds with damage by moth larvae (± SE) on sticky and non-sticky plants of *Bejaria resinosa* in three different populations. Different letters indicate significant differences (Tukey test, significance *P* = 0.05).

Specifically, damage made by florivorous moth larvae was reduced in sticky buds (*Χ*^2^ = 13.693, *df* = 3, *P* = 0.003) and flowers (*Χ*^2^ = 13.423, *df* = 3, *P* = 0.003) with levels of damage varying between populations (buds: *Χ*^2^ = 14.824, *df* = 4, *P* = 0.005; flowers: *Χ*^2^ = 19.717, *df* = 4, *P* < 0.001). However, here we did not observe a significant interaction between the stickiness and the population (buds: *Χ*^2^ = 0.6961, *df* = 2, *P* = 0.7, Fig. 3A; flowers: *Χ*^2^ = 5, *df* = 2, *P* = 0.081, Fig. 3B).

**Figure 4:**
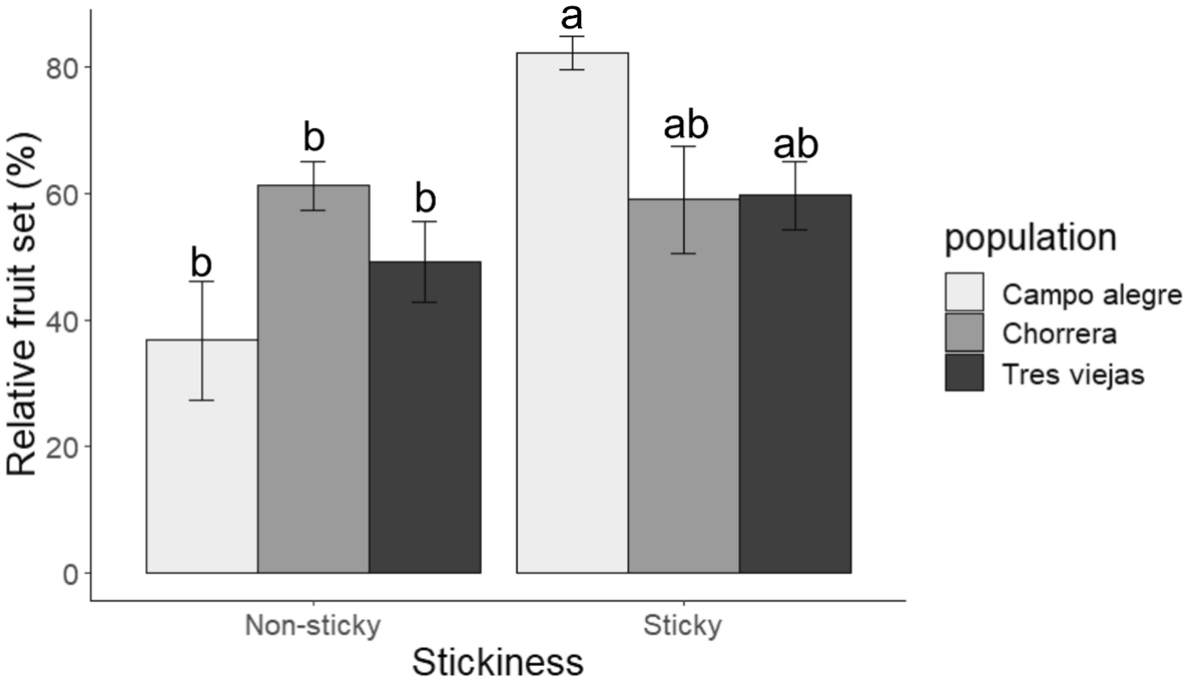
Mean relative fruit set (± SE) of sticky and non-sticky plants of *Bejaria resinosa* in three different populations. Different letters indicate significant differences (Tukey test, significance *P* = 0.05).

Most impactful, floral stickiness was associated with higher fruit set (*Χ*^2^ = 29.511, *df* = 3, *P* < 0.001) linking the negative effects of stickiness on florivory with a positive effect on fitness. As with florivory, fruit set varied with plant population (*Χ*^2^ = 26.89, *df* = 4, *P* < 0.001). In consequence, floral stickiness and fruit set interacted statistically (stickiness × population: *Χ*^2^ = 17.761, *df* = 2, *P* < 0.001, Fig. 4).

### Floral stickiness is causally linked to reduced florivory and increased fruit set in the field

**Figure 5:**
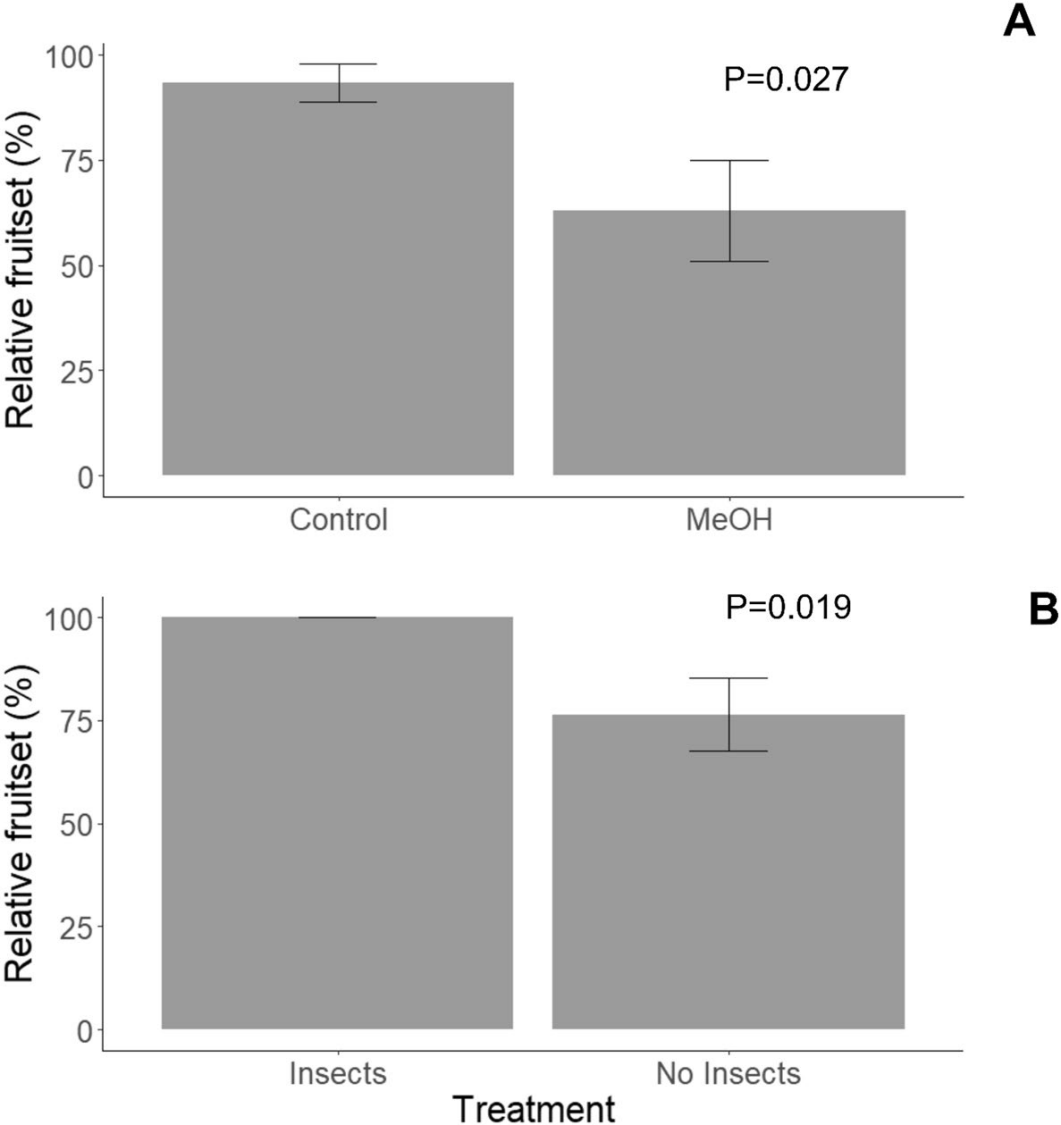
Mean relative fruit set (± SE) of sticky flowers of (**A**) *Bejaria resinosa* washed with 100 μL of 95% methanol (MeOH) or adding methanol to the pedicel (Control) and (**B**) of *Bejaria resinosa* flowers with two different treatments. No Insects treatment, the insects stuck in these flowers were removed manually. Insects treatment, the insects that were removed from the *No Insect* treatment were added to this flower.

In a field experiment manipulating the stickiness of phenotypically sticky flowers, none of the flowers that remained sticky received any herbivore damage, while 21% of the MeOH-washed flowers were attacked by herbivores on average. In consequence, MeOH-washed inflorescences had a 32.5% lower fruit set than non-washed control inflorescences (*X*^2^ = 4.877, *df* = 1, *P* = 0.027, Fig. 5A).

In targeted lab bioassays with generalist herbivores, we found no difference in the petal area consumed by grasshoppers (*Bogotractis varicolor*) between sticky and non-sticky flowers (*W* = 183.5, *P* = 0.6651, Fig. S1A). However, snails (*Helix aspersa*) showed a preference to consume petals from sticky flowers (*W* = 59, *P* = 0.0001, Fig. S1B).

### Number of trapped insects is linked to higher fruit set

The presence of insects on the petals of sticky plants increased the fruit set relative to sticky flowers with no insects (*X*^2^ = 5.4466, *df* = 1, *P* = 0.019, Fig. 5B). We did not observe any kind of damage in either treatment.

### Bird exclusion has no effects on fruit set

**Figure 6:**
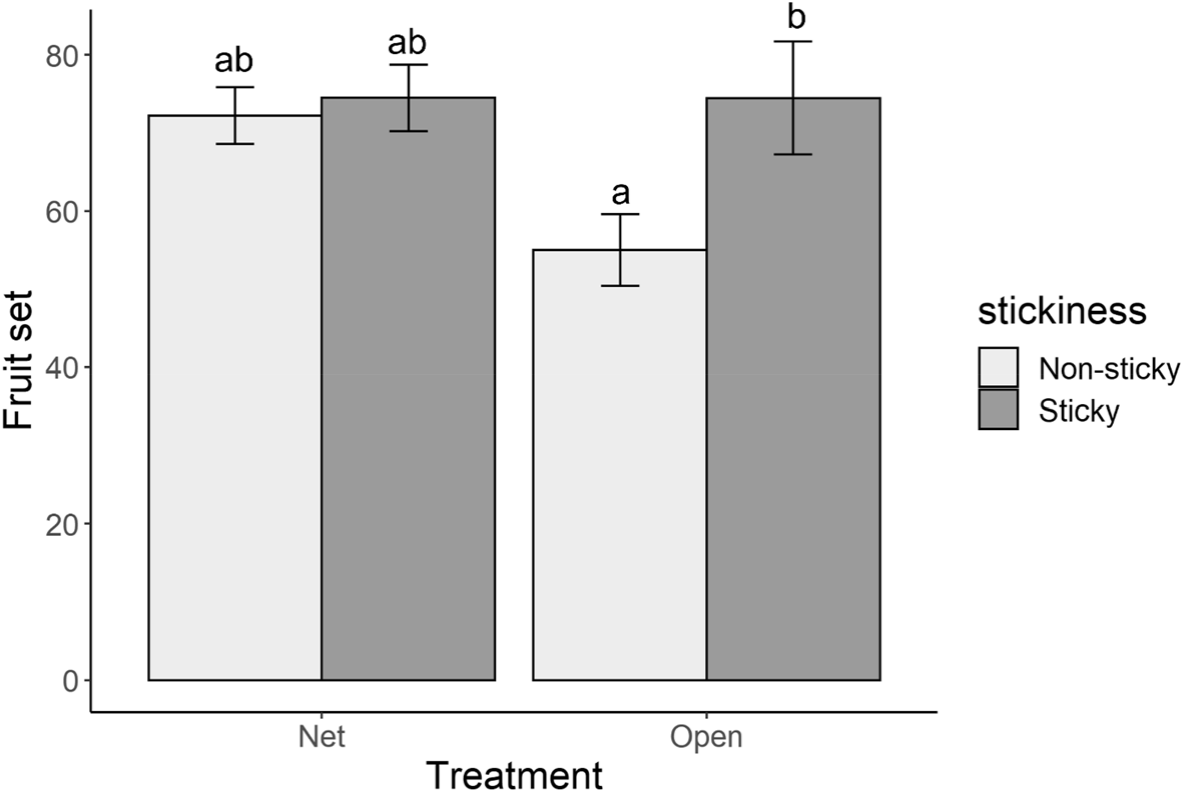
Mean fruit set (± SE) of sticky and non-sticky inflorescences of *Bejaria resinosa*. Some inflorescences were covered by a net that allow bees and bumblebees to visit the flower but exclude hummingbirds and flower pierces, while others remain open to any flower visitor. Different letters indicate significant differences (Tukey test, significance P = 0.05).

The presence of netting to exclude hummingbirds from *B. resinosa* flowers did not affect fruit set (*X*^2^ = 2.1817, *df* = 2, *P* = 0.3359), while stickiness did (*X*^2^ = 5.944, *df* = 2, *P* = 0.051). Nevertheless, there was no interaction between floral stickiness and bird exclusion (*X*^2^ = 1.45, *df* = 2, *P* = 0.22, Fig. 6).

### Non-volatile chemistry differs between stickiness morphs

**Figure 7:**
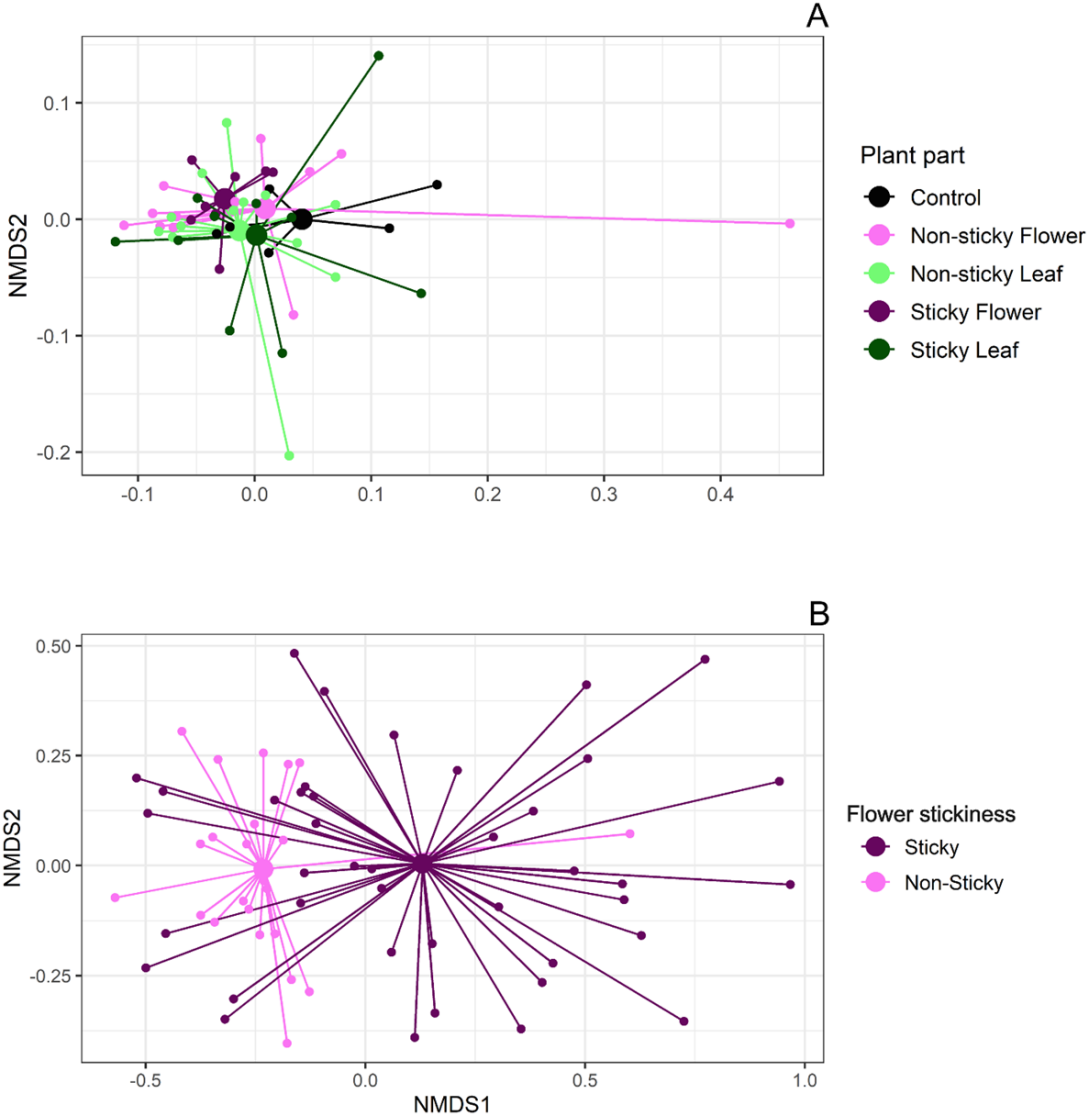
Non-metric multidimensional scaling for (**A**) volatile compounds from flowers and leaves and (**B**) non-volatile compounds from sticky and non-sticky flowers of *Bejaria resinosa.*

In general, emissions of VOCs from flowers and leaves in both sticky and non-sticky plants were low, and there were no detectable differences in VOC composition between flowers and leaves from sticky and non-sticky plants (Permanova: *F*_5,44_ = 0.7163, *P* = 0.6146, Fig. 7A and Fig. S2).

Twelve different non-volatile compounds were identified from petal extracts (Table S1), which made up different chemistry in sticky and non-sticky flowers (PERMANOVA: *F*_1,62_ = 10.864, *P* = 0.001, Fig. 7B). A subsequent random forest analysis (prediction error = 0.1179) revealed two methoxyflavones (methoxyflavone1, methoxyflavone2) and one non-categorized compound as the three components best representing the differences between sticky and non-sticky flowers. Methoxyflavone2 and methoxyflavone1 were found in higher concentrations in the sticky flowers (*t* = − 4.063, *P* < 0.001 and *t* = − 4.083, *P* < 0.001), while the unidentified compound had higher concentrations in the non-sticky plants (*t* = 2.348, *P* = 0.022). The other ten compounds did not differ between sticky and non-sticky flowers.

## Discussion

The production of sticky substances on the surface of leaf tissues, including sepals, has been hypothesized to have a variety of functions, among them: direct defense and indirect defense^32^. Here, we applied this hypothesis framework to floral tissues, which, unlike vegetative tissues, have evolved to mediate interactions with mutualists and are thus prone to expose the conflicts arising from defending against antagonists while still being able to attract mutualists^33,34^. We specifically focused on testing predictions of the direct defense hypothesis and probed for indirect defense hypotheses that sticky substances were expressed on the petal surface. In support of the direct defense hypothesis, we found that stickiness was associated with reduced florivory and could be directly linked to increased reproductive success. Increased fruit set of plants with sticky petals in comparison to those with non-sticky petals was mostly explained by the reduction of general florivory by insects consuming petals as well as by moth caterpillars that consume reproductive structures (e.g. anthers, style, and ovaries). Our data did not exclude the possibility that stickiness does indirectly, e.g. through attraction of predators or fertilization by decomposing insect cadavers stuck on petals, affect plant fitness, which could provide part of the explanation for why the overall link between stickiness and reduced herbivory was context dependent.

In general, our data suggest that stickiness in reproductive tissues can serve the same defensive function as stickiness in vegetative tissues despite the apparent conflict between defending against herbivores and attracting pollinators. Stickiness, in general, can mediate increased direct resistance against insect herbivores by causing insects to get stuck and die, reduce their performance, or repel them from landing on the plant altogether^9,10^. Deterrence was clear in our experiments when flowers from which the stickiness was removed had higher levels of damage compared to wild-type sticky flowers. This deterrence mechanism has been identified for leaf tissues in other plant species. For example, different types of acyl sugars secreted from leaf glandular trichomes of the wild tomato *Solanum pennellii* have been found to function synergistically to deter whiteflies and thrips^35^. Acyl sugars seem to function without apparent toxicity but physically repel or immobilize attackers. Sticky glandular exudates of *Solanum lycopersicum* can trap and starve to death neonate caterpillars of *Helicoverpa armigera*^8^.

**Figure 8:**
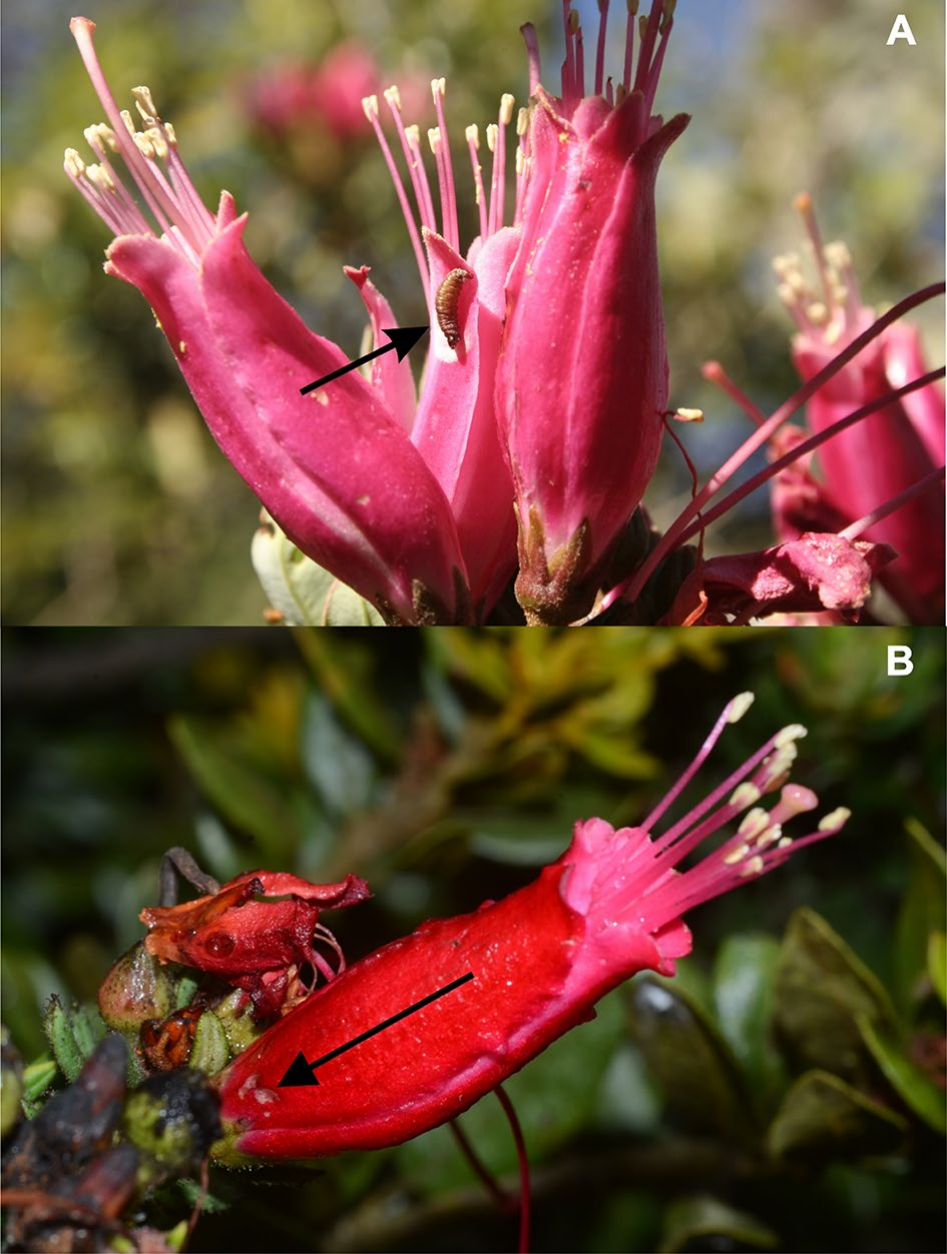
(**A**) Moth larvae trapped on the sticky surface of petals of *Bejaria resinosa.* (**B**) Flower showing the marks left after a visitation by the avian nectar robber *Diglossa* spp.

In *B. resinosa* flowers, larvae of a single Tortricidae species represent the most damaging herbivores because they enter the flower buds and consume all the internal structures. Our data suggest that the physical adhesiveness may keep moths from ovipositing in the first place and caterpillars from moving from flower to flower (Fig. 8A). This observation is consistent with findings on the sticky flowers of *Vriesea bituminosa* (Bromeliacae), where 50% of the trapped insects were phytophagous species^36^, suggesting stickiness as an efficient trait limiting the number of herbivores. For example, the sticky compounds could also be toxic^9,37^ or deterrent^38^. However, among the diverse arthropod community trapped on *V. bituminosa* as well as on *B. resinosa*, are still large numbers of species potentially beneficial to the plants. The resulting conflicting ecological effects are likely factors explaining the population differences in the stickiness effects on plant reproduction and are likely the force maintaining polymorphism in those populations.

This variation could reflect differences in community composition and variable relative impacts of antagonists and mutualists affected by the defensive plant trait. For example, in *Lycopersicon f. glabratum* exudating trichomes reduce predator mobility and also entrap small parasitoids^27^. *Bejaria resinosa* plants likely also suffer from this potential disadvantage. Many predators, such as spiders and parasitoid wasps as well as some potential insect pollinators, such as bees can be seen stuck to the flower petals (Fig. 1). Evaluating this potential ecological cost goes beyond the scope of this paper and requires a different experimental procedure than the one applied here. However, some of the results from our experiments give a clear indication as to where to focus the search for potential fitness trade-offs of stickiness in *B. resinosa*. For example, we applied a very simple field experiment to generally test for the indirect fitness effects of stickiness when insects are stuck to the petals. We found that sticky inflorescences with insects stuck to the petals had a 23.5% higher fruit production than those without insects. The experiment was deemed general (and thus also limited) in its approach because it allows for three mechanistic hypotheses for the apparent indirect effect of stickiness: (A) the trapped arthropods attract predators that then also consume actively feeding florivores (indirect defense), (B) the trapped organisms decompose and provide nutrients to the developing fruits/seeds (protocarnivory), or (C) bird pollinators use the trapped arthropods as an additional nutritional resource and are thus more attracted to these plants^39^ (increased pollinator reward). Because the actual damage levels between flowers with and without insects did not differ, the indirect defense mechanism (hypothesis A) is less likely to be an explanation for the observed fruit set pattern. The increased fruit set was not a result of indirectly reduced florivory. However, we cannot fully dismiss an indirect defense function as a potential mechanism contributing to the population-specific outcomes of stickiness in *B. resinosa*. In other plant species, such as *Aquilegia eximia* (Ranunculaceae) and *Madia elegans* (Asteraceae)^5,13^, the amount of carrion trapped on leaves and stems increased the number of predators attracted to the plants. Also, stickiness increased the efficacy of the predators *Pselliopus spinicollis* and *Hippodamia convergens* on *Heliothodes diminuta* and *Uroleucon madia*^40^. In *B. resinosa*, we consistently observed a yet unidentified hymenopteran species foraging between the corpses on sticky flowers. So, there may be predators taking advantage of dead insects stuck to the plant, as has been seen in *Madia elegans*^13^ and *Nicotiana attenuata*^12^, where the amount of carrion increases the number of predators and guaranteed a higher fruit set.

Similarly, our results do not directly support the hypothesis of a nutritional benefit for the plant from decaying arthropods (hypothesis B) as an explanation for the observed pattern in fruit set. Because of the potential of trapping large numbers of arthropods, stickiness is commonly associated with carnivorous plants such as *Drosera* spp*.* (Droseraceae), *Nephentes* spp. (Nephentaceae), *Triphyophyllum peltatum* (Dioncophyllaceae) and *Pinguicula* spp. (Lentibulariaceae). Accordingly, carnivorous plants are thought to have evolved from plants that expressed some degree of stickiness, with an originally defensive function^41^. When plants become able to absorb some of the nutrients from the corpses, under poor nutrient conditions, it is hypothesized that natural selection can benefit plants with a higher rate of insect trapping and digestive ability^41,42^. In fact, some non-carnivorous plant species (e.g. those that do not produce digestive enzymes) seem to gain nutritional benefits from the trapping of arthropods on otherwise defensive sticky surfaces. Plants like these are often categorized as protocarnivorous^17^. Accordingly, stickiness on floral tissues, like in *B. resinosa*, could potentially provide resources for the developing seeds. The results of our experiment –in which we added insects to flower petals, resulting in increased fruit set- are in support of the hypothesis that *B. resinosa* may gain nutrients from the insect corpses, similar to evidence from insect-trapping *Geranium viscosissimum* leaves and stems^15^. However, while nutrient uptake from decaying animals by leaves is probably more common than currently appreciated, to our knowledge there is no example for a plant being able to take up nutrients through their petals. Alternatively, insects stuck to leaves or flowers have been hypothesized to also be a source of nutrients when the petals fall from the plant and decompose together with the insect cadavers^32^. Again, this later mechanism is not likely to underlie the differences we observed in the manipulative experiment as it may not affect the observed fruit set because there would have been not enough time between anthesis of the manipulated flowers, their decomposition, and eventual uptake back into the plant to affect fruit development and seed growth.

This is leaving interactions with other flower visitors (Hypothesis C), including pollinators as a potential mechanism for how arthropods stuck to the flower petals affect plant fitness. In *B. resinosa* specifically, hummingbirds are the major pollinators in the system and could use insects stuck to the flowers as an additional source of energy and protein. In this case, stickiness could provide a dual function as indirect defense and pollinator reward^39^. Similarly, nectar-robbing *Diglossa* spp. flower piercers could also be attracted by insect cadavers and so mediate indirect defenses.

The hummingbird exclusion experiment showed a reduction in fruit set on the non-sticky inflorescences that were open to the visitation of any pollination mirroring the general differences we found between sticky and non-sticky plants in the other experiments. However, there was no reduction in fruit set when hummingbirds were excluded, rejecting the hypothesis that hummingbirds could be a major indirect factor explaining stickiness-mediated variation between populations. However, this specific result of a relative increase in fitness in non-sticky plants, when birds are excluded, points to yet another potential factor: nectar-robbing flower piercers (*Diglossa* spp.). Our exclusion experiment excluded all birds, including those with potential antagonist effects. As primary robbers, flower piercers could affect plant fitness directly or through facilitating secondary nectar robbers by providing initial access wounds to the flowers (Fig. 8B)^43^, or give better access to florivores. Sticky substances in plants are usually complex compound mixtures^44–46^ with many of the components having some antibiotic, specifically antimicrobial, properties^47^. Thus, the stickiness can provide a mechanism to persistently cover surfaces with antimicrobial substances and so work as a barrier that can prevent secondary infection in the holes that have been opened by other organisms, such as nectar robbers. Given the high prevalence of damage by avian flower piercers in *B. resinosa (29.18% of prevalence)*, stickiness could indeed provide a major mechanism to prevent secondary infections with plant pathogens. The interactions between the plant and other organisms are thought to be largely mediated by plant secondary metabolism. Plant chemistry determines which organisms can interact by providing information before an organism touches the plant tissue (e.g. VOCs, color pigments) and when it touches or manipulates the plant tissue (e.g. toxins, deterrents, detractants, but also VOCs)^48^. The VOC emissions from flowers and leaves of *B. resinosa* are relatively low and not differentiated between tissues and phenotypes. Low floral VOC emissions are common in plants with ornithophilous syndromes of pollination, since the main signal for pollination attraction is not olfactory but visual^49^. Moreover, the similarity in VOC emission between sticky and non-sticky flowers also suggests that *B. resinosa* is not advertising its sticky surfaces to oncoming insects, thus neither repels (chemical aposematism)^50^ nor attracts arthropods to its sticky surfaces (VOC attraction) via VOC cues^51^.

In contrast to the volatile compound production, non-volatile chemistry varied strongly between sticky and non-sticky flowers. The preliminary chemical analysis we present with this study is meant to establish if differences in stickiness between plant phenotypes can be explained by differences in surface chemistry and so begin to close a large knowledge gap on the chemistry of stickiness^41^. The major chemical components we found in far higher concentrations on the surface of sticky *B. resinosa* flowers, methoxyflavones, seem to be commonly associated with sticky plants^47,52,53^ and have reported antifungal activity^47^. Interestingly, experiments with generalist herbivores, in which grasshoppers (*Bogotractis varicolor*) and snails (*Helix aspersa*) were fed on petals in isolation from the plant and thus relatively unaffected by stickiness suggested a higher herbivore resistance to non-sticky floral tissue.

The increased feeding on sticky petals relative to non-sticky petals by the snails is opposite to our field results on herbivory. On one hand, this difference can point to a mainly deterrent, non-toxic nature of the sticky compound mixture on the surfaces of *B. resinosa* petals similar to that mediated by acyl sugars^4,35^ and the potential presence of actually higher concentrations of toxic or anti-digestive compounds in tissues of non-sticky petals. The expression of one not yet identified compound found in the non-sticky petals follows exactly that pattern and could have a deterrent effect on the snails specifically as well as other herbivores. On the other hand, snails may prefer some of the chemistry associated with stickiness. In conjunction with metoxyflavones, terpenes are also very frequent on sticky surfaces^54–56^, and previous studies have found that *H. aspersa* preferentially feeds on terpene-rich tissues^57^. So, it is possible that the stickiness that frequently functions as a defense against most herbivores, in the case of snails is being used as an attractant. In conclusion, the chemical differences in petal surface chemistry observed in this study explain the differences in florivory between the two different stickiness morphs of *B. resinosa*. The understanding of what makes a mixture of compounds sticky, and the physical properties of individual compounds and mixtures goes beyond the scope of this paper. We, however, still try to thoroughly report the chemical differences to move towards that kind of understanding (Table S1).

Although more targeted experiments on the indirect effects of stickiness and the chemical mechanisms are needed, our findings nevertheless indicate that direct and indirect ecological effects of stickiness negotiate the outcome of the conflict between attracting friends while repelling foes. Understanding the ecological conflicts between defending against antagonists while attracting mutualists, specifically with chemical traits in floral tissues, has become a major focus of chemical ecology research in recent years and has been viewed as crucial in understanding the evolution of floral traits^24,58–60^. Tentative indication for the existence of such ecological conflicts in *B. resinosa* is apparent in the context-dependency of the direct defensive effects of stickiness.

## Materials and methods

The study was conducted on two populations of *B. resinosa* in the municipality of Sesquilé: Chorrera (CH), at 2800 m.a.s.l. (5° 2′ 56.51″ N, 73° 46′ 45.90″ W) and Tres Viejas (TV) at 3050 m.a.s.l. (5° 2′ 40.28″ N, 73° 46′ 16.52″ W), and a third population in the municipality of Sopó: Campo Alegre (CA), at 2700 m.a.s.l. (4° 54′ 28.52″ N, 73° 59′ 16.94″ W) in Cundinamarca, Colombia. The flowers of this plant species are visited by numerous species of insects, avian flower piercers (*Diglossa* spp.), and hummingbirds^30,61^. The plant is highly self-compatible but requires pollinator visitation and the pollen has viscin threads that are thought to facilitate pollen transfer^30^.

### Ethics declaration

All of the experiments on intact plants were conducted in natural populations that had no protection status. Small amounts of floral tissue (< 1 g per plant) was removed for lab bioassays and chemical analyses. No leaf tissue was exported outside Colombia and all conduct was performed in accordance to Colombian and international regulations.

### Survey of floral damage and seed set

To determine if the stickiness affects the proportion of damage to flowers by herbivores, we first surveyed sticky and non-sticky plants in each population, then randomly chose and marked one inflorescence per plant to count the number of flowers and buds and the proportion of those with any damage by florivorous species (rate of herbivory). One month later, we recorded the proportion of those flowers that had turned into fruit (fruit set) as a measure of fitness. In total, we recorded data from 50 plants in CH, 38 in TV, and 38 in CA. We found different proportions of sticky and non-sticky plants in the three populations (CH 2:3, TV 5:3, CA 4:1). During the survey, it became evident that much of the damage was due to a single species of Tortricidae (Lepidoptera) larvae eating the anthers, style, and ovules of the flower, usually starting just before the anthesis of the flowers. So, we conducted a second survey to specifically estimate the amount of damage to buds and flowers affected by the caterpillars in sticky and non-sticky plants.

Statistical analyses were done using binomial regression with the total number of flowers or buds and the number of them with damage as dependent variable and stickiness and population as independent factors. Plant ID in each population was used as a random factor. Significance of the factors were assessed by using a likelihood-ratio test. The significance of stickiness as an independent factor was assessed by comparing the complete model (stickiness x population) vs the model that used only population as independent factor. In the same way the significance of the population was tested by comparing the complete model vs the model with stickiness only. Finally, the interaction between stickiness and population was tested by comparing the model with interaction (stickiness × population) vs without interaction (stickiness + population), this was followed by a post hoc test using the CLD function from the ‘emmeans’ in R version 4.1.1^62^.

### Effect of stickiness on fruit set

To test for a causal relationship between stickiness, herbivory, and plant reproduction, we selected two inflorescences on each of ten different plants that produce sticky flowers. Just before anthesis, all of the buds of one of the inflorescences were each washed with 100 μL 95% methanol on a Q-Tip to remove the stickiness (**MeOH treatment)**; the buds on the other inflorescence were kept as a control (**Control treatment)** and 100 μL of MeOH was similarly applied to the pedicels as a sham control for the MeOH treatment. One week after the MeOH application (flowers in full bloom and just beginning to wilt), we assessed floral damage as the number of damaged flowers relative to the total number of flowers in each inflorescence. Inflorescences contained between 2 and 7 flowers. Fruit set was measured as the number of seed pods per initial number of flowers on each infructescence 100 days after the initial treatment. The data were analyzed by using binomial regression using the relative damage and relative fruit set as the response variable, treatment as independent factor, and the individual plant as a random factor. Significance of MeOH washing effects was detected through comparison of models with and without MeOH washing as the independent factor.

In addition to the field experiments, we conducted laboratory feeding assays with two generalist herbivore species not commonly found in the plant to test for the actual feeding resistance rather than stickiness effect in the two different plant morphs.

### Effect of insects trapped on sticky petals on fruit set

To test for a potential proto-carnivorous, indirect defensive, or pollinator-attracting function of stickiness, in the Campo Alegre population, we selected two inflorescences on each of ten different plants producing sticky petals and applied one of two treatments: **No insects treatment**, insects were removed manually from each bud in the inflorescence without removing the stickiness; **Insect treatment**, in this treatment the insects removed in the previous treatment were added for an increased number of insects trapped to each bud. We measured relative damage seven days and relative fruit set 100 days after the initial treatment, as described above. The data were analyzed by using binomial regression using the relative damage and relative fruit set as the response variable, treatment as independent factor, and the individual plant as a random factor.

### Hummingbird exclusion

Based on the findings in the first part of this study, we hypothesized that the population-specific differences in the effects of stickiness on florivory could be due to the differences in the presence of hummingbirds. Sticky plants are efficiently excluding interactions with insects both mutualist and antagonist. Thus, in populations with a high density of hummingbirds such as in the CA population, sticky plants will reap the benefits of protecting their flowers from herbivory without compromising pollination by hummingbirds. In such a population, non-sticky plants will have lower levels of fruit set when florivores are present. On the other hand, in populations with low densities of hummingbirds, sticky plants can be predicted to have a lower rate of pollination due to the additional reduction in pollination by insects. In this case, the costs of not attracting insect pollinators associated with stickiness are predicted to be larger than the benefits of protecting the flowers from florivorous. To test this hypothesis, we chose eight sticky plants and eight non-sticky plants in the Tres Viejas population, and two branches per plant were selected. One of those branches was protected by a net with a mesh of 2 cm, supported on a wire structure that separated the net from the flowers. The holes allowed the major insect pollinators, bees, and bumblebees^30^, to visit the flowers but excluded the hummingbirds. Finally, we measured the relative fruit set of each branch by counting the number of flowers that became fruits on those branches. The results were analyzed by a binomial regression with fruit set as dependent variable. Stickiness and the presence of the net were considered independent factors. The plant was considered a random factor. Effects of the factors were assessed by using a likelihood-ratio test, the significance of stickiness was tested by comparing the complete model (stickiness × presence of the net) vs the model with only presence of the net. In the same way the significance of the presence of the net was tested by comparing the complete model vs the model with only stickiness. Finally, the interaction between stickiness and the net was tested by comparing the model with interaction (stickiness × population) vs without interaction (stickiness + presence of the net), this was followed by a post hoc test using the CLD function from the ‘emmeans’ in R.

### Chemical analysis

We collected volatiles from the headspace of flowers (Sticky n = 8, non-sticky n = 13) and leaves (sticky n = 10, non-sticky n = 13) from plants by enclosing them in 500 mL polyethylene cups fitted with ORBO-32 charcoal adsorbent tubes (Supelco, Bellefonte, PA, USA). Air was pulled through the cup at a flow rate of approximately 150 mL/min for 8 h using an active air sampling vacuum pump (IONTIK, USA). Five additional charcoal tubes collected air from the environment as a control. Tubes were then capped and kept frozen before analysis. Before elution, we added 5 µL tetraline (90 ng/mL) as an internal standard to each tube. The tubes were then desorbed with 350 mL of dichloromethane and samples were analyzed in a Varian CP-3800 GC coupled to a Saturn 2200 MS and fit with a DB-WAX column (J&W Scientific) of 60 m × 0.25 mm id capillary column coated with polyethyleneglycol (0.25 mm film thickness). Helium (99.995%, Airgas^®^) was used as a carrier gas. Total ion chromatograms were integrated, and peak areas of individual compounds were normalized by the area of the internal standard. Tentative identification was made by comparing the mass spectrum with the NIST Mass Spectral Database. As before, we compared the changes in the compositions between sticky and non-sticky flowers and leaves using permutation analysis of variances (PERMANOVA) and a Nonmetric multidimensional scaling (NMDS) on Bray–Curtis distances for visualization.

To analyze the non-volatile corolla surface chemistry, petals from both sticky and non-sticky plants were collected from different plants of those used for other experiments and washed with methanol to remove the sticky layer. These extracts were analyzed on an LC Dionex UltiMate 3000 (Thermo Scientific, Germering, Germany) equipped with a degassing unit, a gradient binary pump, and an autosampler with 120-vial well-plate trays, and a thermostatically controlled column compartment. Chromatographic separation was performed on a Hypersil GOLD aQ C_18_ column (Thermo Scientific, Sunnyvale, CA, USA, 100 mm × 2.1 mm i.d., 1.9 μm particle size). The column temperature was 35 °C, flow rate 0.3 mL/min, injection volume 2.0 μL, the mobile phase was acetonitrile grade LC/MS modified with 0.2% v/v formic acid (A) and an aqueous solution with 0.2% v/v formic acid (B). The initial gradient condition was 100% A, changed linearly to 100% B in 8 min, maintained for 4 min, returned to 100% A in 1 min, and maintained for 3 min. The injection volume was 2 μL. The LC was connected to an Exactive Plus Orbitrap mass spectrometer (Thermo Scientific, Bremen, Germany) with a heated-electrospray ionization (HESI-II) source operated in the positive ion mode. The capillary voltage was set at 3.5 kV. The nebulizer and capillary temperatures were set at 350 and 320 °C, respectively; sheath gas and auxiliary gas (N_2_) were adjusted to 40 and 10 arbitrary units, respectively. Nitrogen (> 99%) was obtained from a generator (NM32LA, Peak Scientific, Scotland, UK). During the full scan MS, the Orbitrap-MS mass-resolution was set at 70,000 (full-width-at-half-maximum, at *m/z* 200, R FWHM) with automatic gain control (AGC) target, 3 × 10^6^, C-trap maximum injection time, 200 ms, and a scan range of *m/z* 100–1000. The ions injected into the HCD-cell via the C-trap were fragmented with stepped-normalized collision energies of 10, 20, 30, and 40 eV. The mass spectra were recorded in the AIF (All-ion fragmentation) mode for each collision energy at R FWHM of 35,000, AGC target, 3 × 10^6^, C-trap injection time, 50 ms, and a mass range of *m/z* 80–1000. The data obtained were analyzed using Thermo Xcalibur 3.1 software (Thermo Scientific, San Jose, CA, USA). Compound identification was based on exact mass measurement ([M]+ or [M + H]+), elemental composition calculation for both protonated molecules and their product ions, and the comparison of retention times of reference substances with those in flower extract´s chromatograms. The metabolomics (METLINTM, http://metlin.scripps.edu) and phytochemistry (PCIDB, http://www.genome.jp/db/pcodb) databases were employed. With the relative peak area data, we compared the changes in the compositions between sticky and non-sticky flowers using permutation analysis of variances (PERMANOVA) and a Nonmetric multidimensional scaling (NMDS) on Bray–Curtis distances for visualization. Moreover, we conducted a random forest (RF) analysis using the packages randomForest^63^ and varSelRF^64^ in R version 3.3.1^62^. To do this, we first conducted an RF classification analysis for sticky and non-sticky flowers. We then used 200 bootstrap iterations to select the compounds that best distinguished between sticky and non-sticky, followed by a t-test with the resulting compounds.

## Supplementary Information

Supplementary Information.

## Data Availability

Raw data are available on Cornell’s eCommons (https://hdl.handle.net/1813/111390) data repository under the title of this publication.
