# Supplementary material for: Defensive functions and potential ecological conflicts of floral stickiness
Source: Sci Rep. 2022 Nov 18;12:19848. doi: 10.1038/s41598-022-23261-2 (PMC9674602; doi:10.1038/s41598-022-23261-2)
Supplement: Supplementary file 1 — Supplementary Information. [file 41598_2022_23261_MOESM1_ESM.docx]

**Supplementary Materials:**

**Resistance mediated by non-sticky traits.**

To test for differences in direct herbivore resistance between sticky and non-sticky plants we conducted laboratory experiments with of two generalist herbivores. We first collected 40 petals from sticky and 40 non-sticky plants, the initial area of those petals was calculated from pictures using the program ImageJ (Schneider, Rasband & Eliceiri 2012). Then, the petals were assigned to either a grass hoper (*Bogotractis varicolor*) or a snail (*Helix aspersa*) contained in a 250 mL clear plastic cup (n=20). After, 12 hours the petals were removed and the final areas of petal remaining was measured again. The areas consumed by each herbivore were analyzed using a Wilcoxon test on R program. As the petals were removed from the flowers and were accessible to the herbivores from the sides, the differences in feeding on the respective petal phenotypes represents a measurement of their direct chemical resistance rather than the deterrent stickiness.


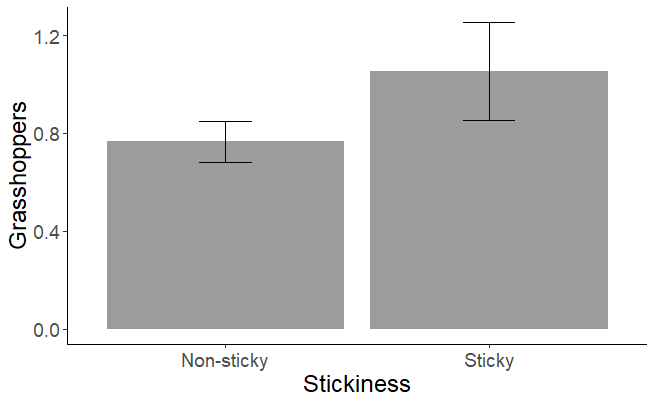


**A**

*P* = 0.6651

Area consumed (cm^2^)


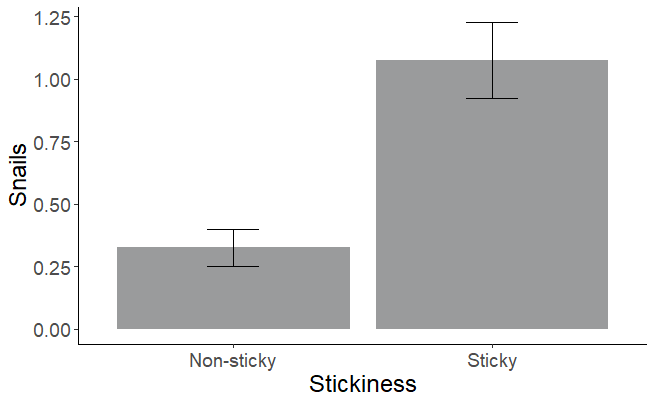


**B**

*P* =0.0001

**Fig S1.** Mean area consumed (± SE) by **A.** grasshoppers (*Bogotractis varicolor*) and **B.** snails (*Helix aspersa*) from sticky and non-sticky petals of *Bejaria resinosa*.

**B**

**A**

5

5

4

4

3

3

2

2

1

1

IS

IS

**Fig S2.** Chromatogram of volatile organic compounds of *Bejaria resinosa* from **A** sticky flowers and **B** non-sticky flowers. Numbers indicate the tentative identification of some of the compounds. **1.** Beta-phelandrene, **2.** 2,6-Dimethyl-1,3,5,7-octatetraene, E,E, **3.** 1(2H)-Naphthalenone, 3,4-dihydro, **4.** 4-Morpholinebutyric acid, .beta.-methyl-.alpha.,.alpha.-dipheyl, **5.** 1-Naphthalenol, 1,2,3,4-tetrahydro**, IS.** Internal standard (Tetraline). No significant differences in composition or quantities were observed between headspaces volatile organic compound emissions of sticky and non-sticky *B. resinosa* plants.

**Table S1**. Tentative identification of non-volatile compounds found in methanol-wash of *Bejaria resinosa* flowers.

| Compound |
| --- |
| Cyanidin-3-glucoside |
| Cyanidin-3-glucoside |
| Quercetin-3-glucoside + Myricetin-rhamnoside |
| Quercitrin+ Kaempferol-glucoside |
| Quercetin 3-arabinoside |
| Quercetin 3-*O*-glucuronide |
| Kaempferol-ramnoside |
| Kaempferol-glucoronide |
| Myricetin 3-*O*-glucoside |
| Methylapigenin |
| Not identified |
| methoxyflavone1 |
| methoxyflavone2 |
